# Targeting cardiac β-adrenergic signaling via GRK2 inhibition for heart failure therapy

**DOI:** 10.3389/fphys.2013.00264

**Published:** 2013-09-26

**Authors:** Alessandro Cannavo, Daniela Liccardo, Walter J. Koch

**Affiliations:** ^1^Center for Translational Medicine, Department of Pharmacology, Temple UniversityPhiladelphia, PA, USA; ^2^Division of Geriatrics, Department of Translational Medical Sciences, Federico II University of NaplesNaples, Italy

**Keywords:** heart failure, β adrenergic system, β blockers, G-protein coupled receptors, G-protein-coupled receptor kinase 2

## Abstract

Cardiac cells, like those of the other tissues, undergo regulation through membrane-bound proteins known as G protein-coupled receptors (GPCRs). β-adrenergic receptors (βARs) are key GPCRs expressed on cardiomyocytes and their role is crucial in cardiac physiology since they regulate inotropic and chronotropic responses of the sympathetic nervous system (SNS). In compromised conditions such as heart failure (HF), chronic βAR hyperstimulation occurs via SNS activation resulting in receptor dysregulation and down-regulation and consequently there is a marked reduction of myocardial inotropic reserve and continued loss of pump function. Data accumulated over the last two decades indicates that a primary culprit in initiating and maintain βAR dysfunction in the injured and stressed heart is GPCR kinase 2 (GRK2), which was originally known as βARK1 (for βAR kinase). GRK2 is up-regulated in the failing heart due to chronic SNS activity and targeting this kinase has emerged as a novel therapeutic strategy in HF. Indeed, its inhibition or genetic deletion in several disparate animal models of HF including a pre-clinical pig model has shown that GRK2 targeting improves functional and morphological parameters of the failing heart. Moreover, non-βAR properties of GRK2 appear to also contribute to its pathological effects and thus, its inhibition will likely complement existing therapies such as βAR blockade. This review will explore recent research regarding GRK2 inhibition; in particular it will focus on the GRK2 inhibitor peptide known as βARKct, which represents new hope in the treatment against HF progression.

## Introduction

Heart failure (HF) is a major and growing public health problem and despite effective therapy outcomes remain poor with a 5-year mortality at 50% (Braunwald, 2001; Hunt, 2005). Clinically, HF is a chronic and severely debilitating syndrome that generally ends up in a vicious cycle of progressive functional decline and it is characterized by the insufficient pumping of blood to meet the needs of the body. During HF, sympathetic nervous system (SNS) activity and levels of catecholamines are increased in an attempt to drive pump function higher. Initially, SNS hyperactivity serves to compensate for the reduced cardiac output, but long-term exposure to high levels of circulating catecholamines causes maladaptive changes to the heart through dysregulation of their β-adrenergic receptor (βAR) targets and also causes myocyte death that leads to maladaptive remodeling of the stressed and failing heart (Woo and Xiao, 2012). Currently, HF therapy is palliative and protecting the heart against SNS bombardment through βAR blockers has shown to offer benefit (Barki-Harrington et al., 2003). However, β-blockers and other neurohormonal blocking strategies are still not ideal therapies as not all patients respond favorably to these agents and thus, new therapeutic strategies are urgently needed. In this regard, a deeper understanding of the underlying molecular mechanisms contributing to the development and progression of the disease represents the best case for future therapeutic advances. In the last two decades, the study of G protein-coupled receptor (GPCR) signaling in failing myocardium has led to the identification of GPCR kinase 2 (GRK2) playing a central role in HF pathology. Accordingly, the inhibition of GRK2 appears to be a powerful therapeutic approach and appears to provide complementation to β-blockade.

### βAR in cardiac physiology and pathology

**Figure 1:**
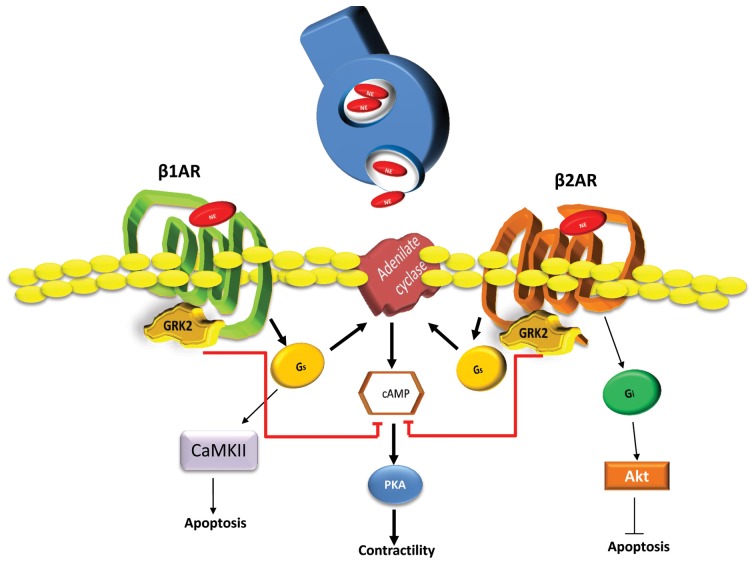
**Differential signaling pathways activated by β_1_ARs and β_2_ARs in cardiomyocytes**. Following SNS catecholamines (Norepinephrine-NE) stimulation, activated β_1_ARs and β_2_ARs induce the production of cAMP through Gs activation of adenylyl cyclase (AC). Next, cAMP activates PKA resulting in positive chronotropic and inotropic effects. Secondarily, β_1_AR through Gs and Calmodulin-dependent kinase (CAMKII) dependent pathway can induce myocyte apoptotic responses after increased catecholamine stimulation. In contrast, β_2_ARs can induce the activation of Gi dependent cell survival and anti-apoptotic signaling through Akt. Both β ARs are regulated by GRK2 phosphorylation causing the dampening of G protein activation and chronically this leads to receptor down-regulation and the loss of inotropic reserve. Heightened GRK2 activity, as seen in human HF, appears to have a net pro-death effect due, at least in part to desensitization of β_2_ARs (Brinks et al., 2010) and also due to novel GPCR-independent mitochondrial targeting (Chen et al., 2013).

βARs are the most important GPCR class expressed in the human heart and represent the most powerful means to increase the pumping function of the heart. In particular, βARs are the prime modulators of heart rate and myocardial contractility in response to catecholamines originating from the SNS (Huang et al., 2011). Three βAR subtypes (β_1_, β_2_, and β_3_) have been identified in human cardiac tissue with the β_1_- and β_2_ARs representing the majority of βARs in the myocyte driving functional responses and these receptors are in a 3–4:1 ratio (β_1_:β_2_) in the normal heart (Frielle et al., 1987; Kobilka et al., 1987; Emorine et al., 1989). The β_3_AR is relatively minor although it is present and may contribute to normal and diseased myocardial regulation (Aragón et al., 2011). Following catecholamine stimulation, both β_1_- and β_2_ARs couple to adenylyl cyclase (AC) stimulatory G protein, Gs, leading to cAMP accumulation within the myocyte and activation of protein kinase A (PKA). This kinase phosphorylates many Ca^2+^ handling protein and some myofilament components leading to positive inotropic, lusitropic and chronotropic effects (Bristow et al., 1990). Importantly, stimulation of cardiac β_2_ARs also causes stimulation of pertussis toxin (PTX)-sensitive Gi signaling pathways (Xiao, 2001). Gi activation can lead to AC inhibition and also other signaling pathways independent of β_1_AR pathways including activation of mitogen-activated protein kinase (MAPK) pathways and also PI3-kinase and Akt pathways (Xiao, 2001). Therefore, these distinct G protein-coupling characteristics of β_1_ARs and β_2_ARs can result in differential regulation and fate of cardiac myocytes (Figure 1). Known differences include myocyte cell death and survival as β_1_AR stimulation leads to apoptosis while β_2_AR signaling favors cell survival pathways (Communal et al., 1999; Zaugg et al., 2000). The later has been shown to be through a β_2_AR-Gi-Gβγ-PI3K-Akt cell survival signaling pathway and the inhibition of this pathway converts β_2_AR signaling from survival to apoptotic (Zhu et al., 2001). β_1_- and β_2_ARs also manifest opposing effects on cardiac cell growth as stimulation of β_1_ARs, but not β_2_ARs, can cause hypertrophy in cultured neonatal and adult rat cardiac myocytes (Schafer et al., 2000; Morisco et al., 2001).

### GRKs

βARs and other GPCRs undergo regulation for signal termination immediately following activation through phosphorylation by a family of kinases known as GPCR kinases (GRKs). GRK2 is a principal GRK involved in intracellular βAR signaling within the cardiac myocytes (Figure 1) and as discussed below, plays a crucial role in HF (Keys and Koch, 2004). Of the seven mammalian GRKs that have been identified to date, GRK2 and GRK5 represent the most abundantly expressed types in the heart and both can regulate βAR signaling (Huang et al., 2011). In addition to βARs, GRK2 and other GRKs phosphorylate many receptors and appear to have some substrate specificity in the heart (Eckhart et al., 2000). All GRKs are serine/threonine kinases with similar structural architecture as they all have a highly-conserved, central catalytic domain (~270 aa), flanked by a variable amino-terminal (NT) domain (~185 aa) and carboxyl-terminal (CT) domain (~105–230 aa) that contains specific regulatory sites (Rockman et al., 1998b; Vinge et al., 2008). The NT-domain harbors several regulatory motifs including a RH domain (regulator of G protein signaling homology domain) and the CT-domain mediates the interactions with lipids and membrane proteins that control the subcellular localization of GRKs (Pitcher et al., 1998; Penela et al., 2010). Importantly, the CT-domain of GRK2 (and the related GRK3) contains a pleckstrin homology domain (PH), which interacts with phosphatidylinositol 4,5-biphosphate (PIP2) and free G_βγ_ subunits. Following these interactions, GRK2 translocates to the plasma membrane enhancing activated GPCRs phosphorylation. GRK5 does not have this domain and does not use G_βγ_ to target the plasma membrane and thus, GRK2 can be selectively targeted through CT-derived peptides that keep GRK2 off the membrane and can limit the desensitization of GPCRs including cardiac βARs (Koch et al., 1993, 1995).

### GRK2 and βAR signaling in HF

As mentioned above, GRKs phosphorylate activated receptors leading to desensitization to control their over-stimulation (Freedman et al., 1995; Rockman et al., 2002) and since βARs are an important part of the heart's response toward stress and injury they are GRK targets. Following GRK-mediated phosphorylation of βARs in the heart, β-arrestins are recruited to these receptors and these adaptor molecules block further G protein activation (Ferguson, 2001). β-arrestins then internalize receptors leading to their degradation, resensitization and return to the membrane or induce G protein-independent signaling (Pitcher et al., 1998). Overall, βAR signaling is critical for both normal and diseased heart function and as introduced above their dysregulation in injured/stressed myocardium is a cardinal characteristic of HF. Over the last two decades GRK2 has been shown to be the central culprit in cardiac βAR dysregulation as its up-regulation causes a loss of βAR responsiveness that is marked by both chronic receptor desensitization and down-regulation, which originally occurs due to heightened SNS activity (Bristow et al., 1982; Iaccarino et al., 1998; Rengo et al., 2009; Raake et al., 2013). Importantly, the major βAR down-regulation that occurs is selective for the β_1_AR and this causes a change in β_1_:β_2_ ratio to closer to equal (Bristow et al., 1990; Rockman et al., 1998a; White et al., 2000). Certainly, the up-regulation of GRK2 (and also probably GRK5) caused by SNS activation acts as a “brake” for cardiac βAR signaling and this is a partial reason why β-blockers have some beneficial effects in HF as they can block the noxious effects of catecholamines on the cardiac myocyte. However, increased GRK2 activity has proven to be maladaptive in HF and its inhibition has emerged as a therapeutic target (Lymperopoulos et al., 2012). Interestingly, chronic β-blocker use has shown including in HF models, to decrease GRK2 expression levels, which from data with GRK2 lowering alone, could contribute to their therapeutic effects (Iaccarino et al., 1998; Rengo et al., 2009).

### Yin and yang of HF therapy-beta adrenergic receptors blockers

β-blockers are currently considered as the mainstay of HF therapy. Behind pharmacologic β-blockade action is the ability of these molecules to inhibit the excessive catecholamine stimulation of inotropic βARs that induce myocyte cell death so resulting in cardiac deterioration, and changes in ventricular mass and that promote ventricular dilation through HF (Mann, 1999; Bristow, 2011). Importantly, although β-blockers only indirectly address key molecular signaling alterations within the cardiac myocyte, their clinical use substantially improves HF prognosis increasing survival rate of HF patients and reducing re-hospitalization (Bristow, 1997, 2000). Interestingly, it has been observed that at molecular level, sustained therapy with β-blockers in HF is associated with resensitization of βARs, normalization of GRK2 levels and activity and as a consequence, β-blockers cause the upregulation of cardiac βARs (down-regulated in HF) increasing βAR signaling when they are activated (Leineweber et al., 2005). However, the full extent of whether these molecular changes are the true therapeutic mechanisms of action of βAR antagonists in HF is not fully understood as there are some specific differences in β-blockers classes used. In fact, β-blockers are a heterogeneous group of pharmacologic agents with a number of different actions that include β_1_AR and β_2_AR antagonism, intrinsic sympathomimetic activity (ISA) (Jaillon, 1990), inverse agonism (Chidiac et al., 1994) and guanine nucleotide binding regulation (Bristow et al., 1992). Further, β-blockers may possess additional properties such as vasodilatation via α_1_AR antagonism and non-adrenergic pharmacological properties such as quinidine-like (or “membrane stabilizing”) effects in model systems (Tritthart et al., 1969) and may have also significant antioxidant properties (Dandona et al., 2000).

Of note, β-blocker therapy has limitations in the HF patient population and it is not tolerated by all patients and is certainly not an ideal therapeutic. For example, long-term treatment with β-blockers in HF patients is associated with risk of unwanted systemic side effects. In this context it has been described that increased bradycardia (due to depression of the cardiac conduction system), hypotension, dizziness, weakness, and worsening of depression represent some of the major deleterious effects of β-blocker non-selectivity (Packer, 2001). Furthermore, dose, a critical factor for therapeutic success, has to be titrated individually for each HF patient. Finally, not all HF patients are suited for β-blocker treatment, opening a demand for new therapeutic strategies. For this reason the development of new potential molecules that minimize the unfavorable effects and potentially allow dose reduction is absolutely needed and we believe inhibiting GRK2 is an attractive target not only because it can also normalize βAR signaling but also it appears to have exciting and novel non-GPCR effects that has appeal for targeting its inhibition that can synergize with β-blocker use in HF patients (see below).

### GRK2 non-receptor functions in HF

Although as discussed above it is clear that enhanced activity of GRK2 in HF negatively affect βAR signaling, we have recently demonstrated that GRK2 can induce myocardial pathology through other systems including non-GPCR activity that can negatively affect myocyte metabolism and cell survival (Brinks et al., 2010; Ciccarelli et al., 2011; Chen et al., 2013; Fan et al., 2013). For example, data now supports GRK2 being a molecular link between the excessive neurohormonal activation that follows cardiac stress and initiation of defects in myocyte energy substrate use by negatively affecting glucose uptake in the myocyte through insulin-dependent phosphorylation of insulin receptor substrate-1 (IRS1) that causes a loss of signaling (Ciccarelli et al., 2011). Thus, GRK2 appears to directly modulate signaling through this non-GPCR and participates in the physiological regulation of myocardium insulin signaling. Moreover, our group have also showed that elevated levels of GRK2 in cardiomyocytes cause excessive cell death after acute ischemic injury and targeted inhibition or genetic deletion significantly protects the heart (Brinks et al., 2010; Fan et al., 2013), which importantly argues against GRK2-up-regulation being initially adaptive as elevated GRK2 activity appears to be maladaptive at all times prior to and after cardiac stress. The pro-death effects of GRK2 may be β_2_AR dependent (Brinks et al., 2010) or non-GPCR dependent as we have found that myocyte death during ischemic stress can be regulated by GRK2 levels found within mitochondria as GRK2 is associated with increased mitochondrial-dependent pro-death signaling and also caused increased Ca^2+^-induced opening of the mitochondrial permeability transition pore, a key step in cellular injury (Chen et al., 2013). Accordingly, GRK2 inhibition reduces IRS1-phosphorylation improving insulin signaling and as a result increase glucose uptake in the ischemic cardiomyocytes and its inhibition but also reduces the pro-apoptotic pathway activated by this kinase in response to ischemia. For these reasons it is important to underline that these non-classical roles of GRK2 strongly support the idea that developing a therapy that selectively inhibits GRK2 could synergize and increase the efficacy of β-blockers as GRK2 inhibition would result in additional benefits independent of βARs. In fact, several animal studies have shown significant HF benefit when GRK2 is lowered or inhibited concurrent with βAR antagonists (Harding et al., 2001; Raake et al., 2008; Rengo et al., 2009).

### GRK2 inhibition as a potential approach for HF treatment

Over the last two decades, our lab and others have shown that lowering GRK2 expression or activity in the injured, stressed or failing heart can prevent or reverse ventricular dysfunction at the functional and morphological level (Huang et al., 2011). This has been shown using a CT-derived peptide inhibitor known as the βARKct and also in GRK2 knockout (KO) mice. First, cardiac-specific βARKct transgenic mice were created showing increased inotropic reserve (Koch et al., 1993) and these mice have been used to prevent HF in several genetic mouse models (Rockman et al., 1998a; Freeman et al., 2001; Harding et al., 2001). In addition, viral-mediated βARKct delivery to rats, rabbits, and pigs have shown significant beneficial effects including improved cardiac function and reverse ventricular remodeling (White et al., 2000; Shah et al., 2001; Tevaearai et al., 2001; Rengo et al., 2009; Raake et al., 2013). Similar HF rescue by induced KO of GRK2 in mice after HF was evident is consistent with βARKct-mediated activity being GRK2 inhibition (Raake et al., 2008). Although there are some minor differences between GRK2 inhibition with βARKct and GRK2 KO (Matkovich et al., 2006; Raake et al., 2008; Völkers et al., 2011) the data is overwhelming in supporting GRK2 targeting as beneficial in the failing heart.

Importantly, a recent pre-clinical, large animal HF study has been done that demonstrates the clinical potential of βARKct-mediated gene therapy (Raake et al., 2013). This study used adeno-associated virus serotype-6 (AAV6) to deliver βARKct to a post-ischemic HF model in the pig (Pleger et al., 2011; Raake et al., 2013). We found that AAV6-βARKct delivery via retrograde coronary venous perfusion ameliorated LV function, and suppressed adverse cardiac remodeling and fetal gene expression in this model (Raake et al., 2013). Of note, this pig study recapitulated similar results found with AAV6-βARKct delivery to a rat model of HF where it was also shown that βARKct worked significantly better that β-blockade alone and the two were complementary together (Rengo et al., 2009). Of importance, both of these studies showed that chronic GRK2 inhibition results in significant lowering of catecholamines and aldosterone demonstrating feedback to decrease the neurohormonal outflow associated with negative prognosis in HF (Rengo et al., 2009; Raake et al., 2013). These two studies in particular are important as they show reversal of the disease process and the pig study closely reflect human pathophysiology and is a pre-requisite for future clinical trials.

### Emerging small molecule inhibitors of GRK2

Gene therapy for HF is now becoming a reality (Jaski et al., 2009; Jessup et al., 2011) and AAV6-βARKct trials are in the planning stages, GRK2 inhibition by small pharmacological agents would offer many advantages to the HF patient. Interestingly, several recent molecules have been developed and described that have GRK2 inhibitory properties. Two decades ago, heparin and related compounds were shown to block GRK2 activity however, the direct access to GRK2, the high concentration and the intrinsic cytotoxicity made them not useful for either in cell-based assays or in *in vivo* scenarios (Lohse et al., 1989; Kim et al., 1993; Hasbi et al., 2000; Kassack et al., 2000; Winstel et al., 2005). RNA molecules such as aptamers have been also investigated as a new approach to efficiently block GRK2 activity and the RNA-aptamer C13, was shown to be able to bind to GRK2 with a high affinity and inhibit GRK2-catalyzed rhodopsin phosphorylation (Mayer et al., 2008). All the studies that have analyzed the efficacy of C13, suggest that this RNA-aptamer might represent a starting point for the development of small molecules that specifically target GRK2. Unfortunately, this molecule has been tested only in *in vitro* models and no *in vivo* study at this time is present.

Interestingly, molecules have recently emerged that target the GRK2-G_βγ_ protein-protein interaction and thus, have mechanisms identical to the βARKct. M119 is such a molecule (Bonacci et al., 2006; Casey et al., 2010) and it has been shown to work *in vitro* and *in vivo* on cardiac cells and in the heart preventing ventricular dysfunction after chronic catecholamine exposure and also showing positive results similar to the βARKct in a genetic model of cardiomyopathy (Casey et al., 2010). Gallein is a related molecule that blocks GRK2-Gβγ and it has also shown positive results *in vivo* (Piao et al., 2012). These are promising results, however, these compounds are not true pharmacological agents in a “drugable” sense and have severe limitations that preclude human use (Casey et al., 2010).

Recently, we have found that an existing FDA-approved drug has significant GRK2 inhibitory properties and potentially this off-target effect may be seen in humans. The serotonin reuptake inhibitor (SSRI), paroxetine has affinity for GRK2 and has significant GRK2 inhibitory properties *in vitro* and *in vivo* (Thal et al., 2012). Paroxetine binds in the active site of GRK2 and stabilizes the kinase domain in a novel conformation in which a unique regulatory loop forms part of the ligand binding site (Thal et al., 2012). Further, this drug causes increased isoproterenol-induced shortening and contraction amplitude in cardiomyocytes *in vitro*, and pretreatment *in vivo* of mice with paroxetine before isoproterenol significantly increases left ventricular inotropic reserve with no significant effect on heart rate (Thal et al., 2012). This agent used for clinical depression probably is not viable for use as a specific GRK2 inhibitor but is a great starting point for chemistry to develop novel GRK2 inhibitors that can be used eventually for cardiovascular disorders.

## Conclusions and future perspectives

**Figure 2:**
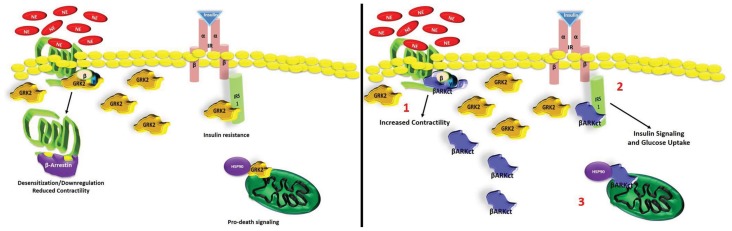
**Multiple protective role of βARKct in failing myocardium against high GRK2 levels**. Schematic representation of GRK2-inhibition mediated by βARKct: (1) βARKct, like GRK2, binds to G_βγ_ subunits after GPCR (e.g., βARs) activation and reduces the capability of GRK2 to induce dysregulation/downregulation of these receptors; (2) βARKct antagonizes GRK2 dependent phosphorylation of IRS1 increasing glucose uptake in myocytes; (3) βARKct blocks the ischemia-induced mitochondria localization of GRK2 through inhibition of MAPK-dependent Hsp90 binding, inhibiting pro-apoptotic signaling.

As demonstrated by us and others, targeted GRK2 inhibition primarily by βARKct expression and some emerging small molecules have shown sustained improvement of global cardiac function and reversal of LV remodeling at least in part due to the normalization of the neurohormonal signaling axis and βAR signaling. In addition, non-GPCR effects of lowering GRK2 activity, has positive effects on cardiac metabolism and on cell survival/death pathways (Figure 2). Importantly, the specific targeting of GRK2 appears similar whether there is inhibition by βARKct or deleting gene expression. Therefore, taken together, these results strongly suggest that the inhibition/lowering of GRK2 activity is a valid and promising novel molecular approach for treating HF. Most studies, including studies with βARKct expression in HF pigs, have shown a reversal of βAR dysfunction including receptor upregulation and a normalization of signaling; however, no doubt, there are effects of the βARKct that go beyond resensitization of cardiac βARs and these effects are currently being explored by us and others. Therefore, these results have launched a clinical gene therapy approach using the βARKct with a Phase I clinical trial being actively planned in order to obtain the best cardio-selective HF treatment that, as widely proven by all gathered data collected by us, could be used alone or in conjunction with the actual β-blockers therapy.

### Conflict of interest statement

The authors declare that the research was conducted in the absence of any commercial or financial relationships that could be construed as a potential conflict of interest.
